# Osteoid Osteoma: A Unique Presentation in a Child's Lesser Toe

**DOI:** 10.1155/2021/8876584

**Published:** 2021-07-27

**Authors:** Michel Bellemans, Nicolas de Saint-Aubin de Somerhausen, Phu Quoc Lê

**Affiliations:** ^1^Hôpital Universitaire des Enfants Reine Fabiola, Brussels, Belgium; ^2^Institut Universitaire Jules Bordet, Brussels, Belgium

## Abstract

**Introduction:**

Osteoid osteoma is an uncommon, small, benign, self-limiting, and usually painful tumor of the skeleton. Diagnosis can be straightforward if seen in the usual locations as the femur and the tibia in young adults, who present with nocturnal pain, alleviated by salicylates. The diagnosis can be more challenging in the spine, pelvis, hand, or feet. *Case Report*. We report the case of an 11-year-old boy who was treated symptomatically for a painful toe since 10 months, without a definitive diagnosis. X-ray, MRI, and scintigraphy, along with the typical nocturnal pain and swelling of the toe, suggested an osteoid osteoma, confirmed by histology after excisional biopsy of the lesion.

**Conclusion:**

Osteoid osteoma should always be included in the differential diagnosis when it comes to nocturnal pain without systemic signs, even in unusual places in children. The awareness should lead to a prompt diagnosis and treatment.

## 1. Introduction

Jaffe [1] is accredited with the first description of this small, benign, and painful bone tumor. The tumor is osteoblastic of nature and produces prostaglandins [2], explaining its response to nonsteroidal anti-inflammatory drugs, especially salicylates. The tumor accounts for 10% of all benign bone tumors. It is more frequent in the second and third decade, with a predilection for the femur and tibia [3].

Definite diagnosis is usually based on the typical clinical presentation, high uptake on Tc-99 m scintigraphy, and the finding of the nidus, surrounded by sclerotic bone on X-ray or CT scan. MRI shows the lesion, surrounded by an inflammatory reaction on T2-weighted images or STIR [4].

Differential diagnosis should include infection such as subacute osteomyelitis, tuberculous spina ventosa, or malignant tumoral disease like Ewing sarcoma [5–9].

Treatment is usually obtained by destroying the cellular components of the nidus by means of heating with a radiofrequency probe under radiologic control in a percutaneous manner [10]. The drawback of this technique is the absence of histologic confirmation. In less typical presentations or in the proximity of delicate structures, an excisional and curative biopsy can be performed.

Involvement of the distal phalanx of a lesser toe is exceptional. The few cases in the literature report a painful soft tissue swelling of the distal aspect of the toe with clubbing and overgrowth of the nail. To our knowledge, this is the first case described in a child.

## 2. Case Report

An 11-year-old boy was referred to our pediatric rheumatologic reference center with a history of swelling and permanent pain of the distal end of his fourth toe without know trauma since 10 months. He presented no systemic symptoms, but the lab finding showed an increase in antinuclear antibodies without an increase of the general inflammatory parameters.

He presented a partial response to ibuprofen and was treated symptomatically with a plaster cast without beneficial result.

Psychologic counseling was offered since the boy was very saddened because he could no longer participate in sport activities like soccer.

A fine needle biopsy was performed: no germs were found and there was not enough tissue to permit the histologic examination.

The Tc-99m scintigraphy showed an increased uptake of the fourth toe, revealing an intense osteoblastic activity.

**Figure 1:**
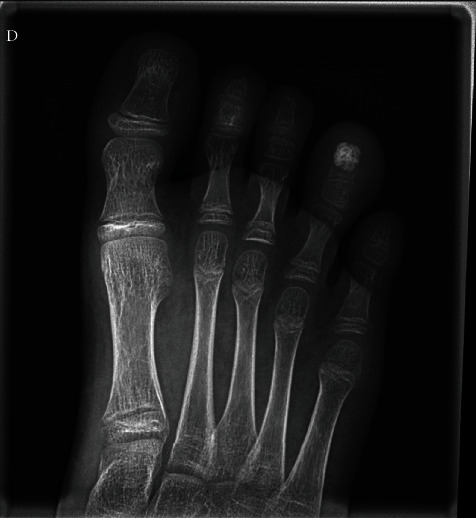
X-ray demonstrating the large nidus and the preservation of the proximal epiphysis. The important swelling of the fourth toe is well demonstrated.

A new X-ray was performed, revealing a partial sclerotic a slightly lobulated mass, replacing two-thirds of the distal phalanx. The mass was delineated with a radiolucent border from the remaining metaphyseal region. The physis remained intact (Figure 1).

**Figure 2:**
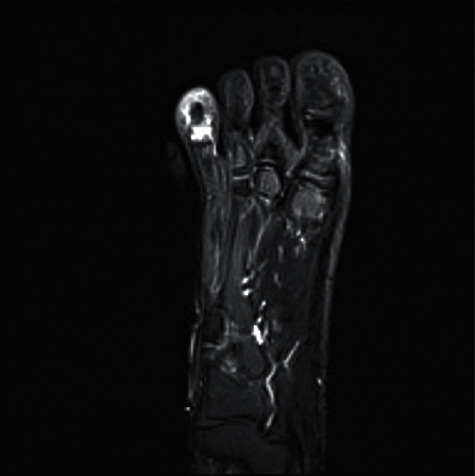
T2-weighted image on MRI, showing the sclerotic nidus hyposignal and the surrounding inflammation of the soft tissues around (hypersignal).

**Figure 3:**
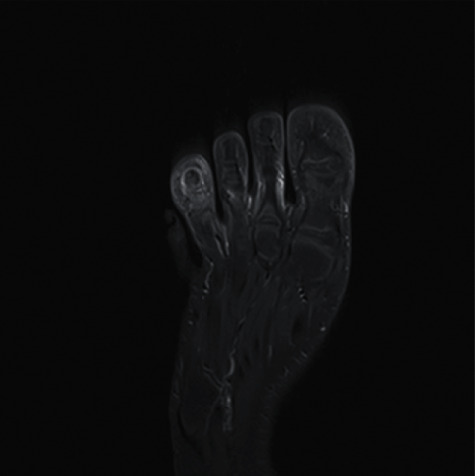
Gadolinium-enhanced T1-weighted images on MRI, showing the relative avascular nidus surrounded by hypervascular inflamed tissues.

The MRI showed a hyposignal center of the distal end of the third phalanx of the fourth toe with surrounding inflammatory response and edema (Figure 2). The injection of gadolinium shows an increase of the signal in T1 around a more avascular center (Figure 3).

**Figure 4:**
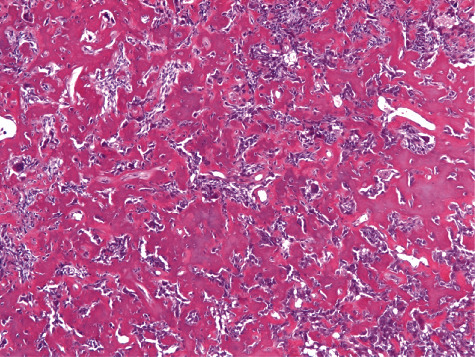
Histologic analysis demonstrating the characteristic pink-staining osteoid in a fibrovascular stroma (H&E staining, ×300).

We decided to perform an excisional biopsy. We found a grayish, slightly lobulated, and rather hard mass, which popped out in toto after the incision. The specimen was sent as such for histologic examination and the diagnosis was confirmed (Figure 4).

The defect was filled with lyophilized bone and the skin closed.

**Figure 5:**
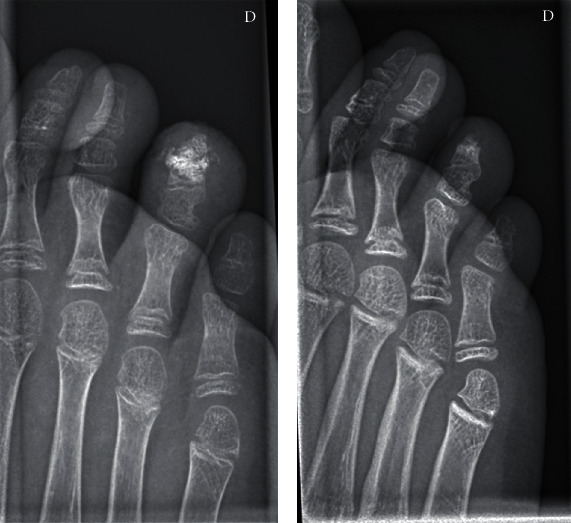
(a) X-ray demonstrating the lyophilized bone graft three week after the intervention. (b) X-ray demonstrating the complete incorporation and remodeling of the graft one year after the intervention.

The day after the excision the patient reported immediate relief and three weeks later, he resumed sports activities. After one year, the swelling of the toe subsided totally and the patient remained asymptomatic (Figures 5(a) and 5(b)).

## 3. Discussion

This report illustrates the diagnostic difficulties and its consequences when it comes to atypical presentations of uncommon pathologies. The few cases reported in the literature all concern adults and all presented with a nidus, surrounded by sclerotic bone. In our case, no sclerotic bone is found surrounding the large nidus which has replaced the distal two-thirds of the distal phalanx. The MRI findings are in contradiction with the notion of a high vascularity in the nidus reported by several authors. On the contrary, the perinidal tissues appear clearly hypervascular with the presence of inflammatory edema.

## 4. Conclusion

To our knowledge, an osteoid osteoma in the third phalanx of a lesser toe in a child was never reported in the literature. This fact, together with a high titer of antinuclear bodies, led to a differential diagnosis of arthritis versus infectious disease, even in the absence of a rise in inflammatory markers, and caused a substantial delay in diagnosis a concomitant treatment.

## 5. Clinical Message

We hope that this case report will raise the awareness of the existence of osteoid osteoma in children, even in atypical locations.

## Data Availability

No data were used to support this study.
